# Oxidative changes in the blood and serum albumin differentiate rats with monoarthritis and polyarthritis

**DOI:** 10.1186/s40064-016-1671-1

**Published:** 2016-01-15

**Authors:** Adelar Bracht, Sandra Silva Silveira, Cristiane Vizioli Castro-Ghizoni, Anacharis Babeto Sá-Nakanishi, Márcia Rosângela Neves Oliveira, Ciomar Aparecida Bersani-Amado, Rosane Marina Peralta, Jurandir Fernando Comar

**Affiliations:** Department of Biochemistry, State University of Maringá, Maringá, 87020900 Brazil; Department of Clinical Analysis, State University of Maringá, Maringá, 87020900 Brazil; Department of Pharmacology and Therapeutics, State University of Maringá, Maringá, 87020900 Brazil

**Keywords:** Chronic inflammation, Adjuvant-induced arthritis, Antioxidant status, Serum albumin oxidation

## Abstract

Adjuvant arthritis in rats, as rheumatoid arthritis in humans, may be of greater or lesser severity, namely polyarthritis and monoarthritis, respectively. The present study was planned to evaluate the oxidative changes in the blood and specifically in the serum albumin of rats with adjuvant-induced mono- and poly-arthritis. Total antioxidant capacity, thiols, carbonyl groups, albumin, uric acid and ascorbic acid were measured in the total serum. The specific oxidative status of albumin was also measured after separation by affinity chromatography. All serum oxidative parameters were close to normal in monoarthritic rats with the exception of the ascorbic acid concentration, which was 23 % lower, and albumin carbonyl groups, which were 64 % higher. Many modifications were found in polyarthritic rats, specially the ascorbic acid concentration (35 % lower) and albumin carbonyl groups (102 % higher). The results revealed that the levels of ascorbic acid in the serum and carbonyl groups in the albumin molecule can be regarded as indicators of the severity of arthritis since they were modified by both monoarthritis and polyarthritis, but to different degrees.

## Background

Rheumatoid arthritis occurs in 0.5–1.0 % of the adult population worldwide and in addition to the osteoarticular manifestations it is associated with an increased mortality rate, mainly due to cardiovascular complications (Uhlig et al. 2014; Kitas and Gabriel 2011; Gabriel and Michaud 2009). The pathophysiology of arthritis involves an intense hyperplasia of the articular cartilage with participation of proinflammatory cytokines and overproduction of reactive species in the synovium, such as superoxide anion $({\text{O}}_{2}^{ - } \cdot ),$ hydroxyl radical $({\text{HO}} \cdot ),$ hydrogen peroxide (H_2_O_2_), and others, which act as mediators of tissue injury (Misko et al. 2013; Kundu et al. 2012; Filippin et al. 2008; Halliwell and Gutteridge 2007). Rheumatoid arthritis is a systemic disease and in addition to the joints other organs are affected (Mcinnes and Schett 2011; Sattar et al. 2003). The oxidative status is likewise changed in the serum blood of patients with rheumatoid arthritis and also in the liver, brain and vascular tissue of rats with adjuvant arthritis (Sarban et al. 2005; Kamanli et al. 2004; Lemarechal et al. 2006; Vasanthi et al. 2009; Seven et al. 2008; Taysi et al. 2002; Wendt et al. 2015; Comar et al. 2013; Haruna et al. 2007).

Rheumatoid arthritis may have a variable evolution, ranging from a mild and intermittent form to another more severe and progressive (Ohrndorf and Backhaus 2013). Similarly, periods of crisis (active arthritis) and remissions (inactive arthritis) are typical (Peluso et al. 2011). Several efforts have been made to correlate the oxidative state of blood and synovial fluid with the severity of the symptoms, activity of the disease, or even mortality rate (Datta et al. 2014; Stamp et al. 2012; Montecucco and Mach 2009; Nicholls and Hazen 2005). Although the reactive species are unstable, antioxidants and oxidation-modified proteins in the serum are more stable and may be a useful tool to correlate the serum oxidative status with the activity or severity of rheumatoid arthritis.

Carbonylation and nitration of amino acids are the most common oxidative modifications in serum proteins and uric acid, albumin and ascorbic acid are the major antioxidants components of the serum (Pavone et al. 2011; Matsuyama et al. 2009; Wayenberg et al. 2009; Kaur and Halliwell 1994; Turell et al. 2013; Yeum et al. 2004). Albumin alone accounts for approximately 70 % of the serum antioxidant capacity (Taverna et al. 2013), which is attributed mainly to the sulfhydryl group (thiol) of cysteine 34 (Cys-34), the only thiol group of this protein (Fanali et al. 2012). About two-thirds of serum albumin contain the thiol group of Cys-34 in the reduced form, which correspond to approximately 80 % of all serum thiol groups (Roche et al. 2008). Furthermore, albumin corresponds to more than 50 % of all serum proteins and it is the only one that shows significant antioxidant activity (Taverna et al. 2013). In rheumatoid arthritis, the serum albumin thiol is more oxidized in arthritic patients than in healthy volunteers (Lemarechal et al. 2006; Banford et al. 1982) and the serum albumin levels fall in proportion to the severity of the disease (Cylmik et al. 2010).

Considering the oxidative changes in serum proteins, particularly albumin, and serum antioxidants in rheumatoid arthritis, as mentioned above, it is possible that the extension of these changes reflects different activities of the disease. Taking these hypotheses into consideration, the present work was planned to evaluate the oxidative status of both the serum and the albumin fraction of rats with adjuvant-induced arthritis with different degrees of severity. The latter is an experimental immunopathology in rats which shares many features of rheumatoid arthritis in humans and also presents prominent oxidative changes in the serum and synovial fluids of the animals (Pearson and Wood 1963; Bendele et al. 1999; Szekanecz et al. 2000; Arab and El-Sawalhi 2013; Nemirovskiy et al. 2009; Kogan et al. 2005).

Like rheumatoid arthritis, the adjuvant arthritis may also be of greater or lesser severity, depending on the dose of the adjuvant that is utilized inthe induction. The classical model, named polyarthritis, is induced by high doses of Freund’s adjuvant and shows a strong and generalized inflammatory response, which affects multiple joints (five or more joints) (Donaldson et al. 1993; Bendele et al. 1999). On the other hand, it is also possible to induce a milder arthritis, known as monoarthritis, which is induced by a smaller dose of adjuvant and presents minor systemic effects and affects only one joint (Bracht et al. 2012; Donaldson et al. 1993). Thus, the present study aims to provide a detailed picture about the oxidative changes in the serum of rats with monoarthritis and polyarthritis and should also allow extrapolations for the serum of patients with greater or lesser severity of rheumatoid arthritis.

## Results

### Characterization of the experimental model

**Fig. 1:**
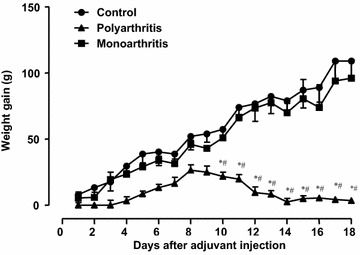
Effects of Freund’ s adjuvant injection in Holtzman rats on body weight gain. Polyarthritis and monoarthritis were induced in rats as described in “Methods” section. The weights of the rats were monitored daily for 18 days after adjuvant injection. *Each point* represents the mean ± SE of the mean of 3–4 animals. *p < 0.05 indicates statistical difference from the controls and ^#^p < 0.05 from the monoarthritic condition

The weight of rats was monitored daily during a 18-day period after adjuvant injection and the weight gain is shown in Fig. 1. The mean of the initial weight of animals was 202 ± 4.2 g. The weight gain of monoarthritic rats was not different from that of the controls. Polyarthritic animals gained weight only slightly and initially, having returned to the same initial body weight at day 18 (195 ± 8.8 g).

**Fig. 2:**
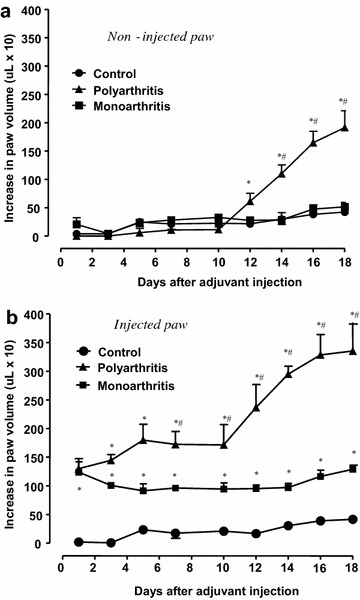
Development of the inflammatory response to Freund’s adjuvant in the posterior non-injected paw (**a**) and injected paw (**b**). Adjuvant arthritis was induced by injection of 0.1 ml of Freund’s adjuvant (inactivated *M. tuberculosis*) into the left hind paw. The concentrations of the injected suspension were 5.0 mg ml^−1^ for polyarthritis induction and 1.0 mg ml^−1^ for monoarthritis induction, as described in the “Methods” section. The volume of the paws was monitored daily by plethysmography and the results are expressed as the increase in paw volume. *Each point* represents the mean ± SE of the mean of 3–4 animals. *p < 0.05 indicates statistical difference from the controls and ^#^p < 0.05 from the monoarthritic condition

The development of inflammation was assessed by measuring the edema formation in the posterior paws. The results are shown in Fig. 2. The initial volume of the hind paw before the injection was 1.35 ± 0.05 ml. An inflammatory reaction in the injected paw was observed on the first day post-adjuvant injection for both mono- and poly-arthritic rats (+90 %; panel a). This increase in the paw volume remained constant until the end of the experimental period (18 day) for monoarthritis. On the other hand, the volume of the injected paw of polyarthritic rats further increased progressively and on day 18 it was 81 % greater than that of monoarthritic rats. The volume of the non-injected paw of both controls and monoarthritic animals did not change during the 18 days period (Fig. 2b). However, the inflammatory response in the non-injected paw started at day 10 for polyarthritic rats and progressed until day 18 (+84 % compared to controls).

### Profile of serum proteins

**Fig. 3:**
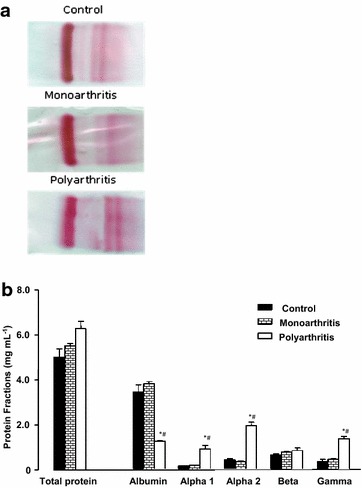
Fractionation of the serum proteins by cellulose acetate electrophoresis. Cellulose acetate electrophoresis was performed with serum obtained from controls, mono- and poly-arthritic rats, as described in the “Methods” section. **a** Typical sample of dried electrophoresis strips for each experimental condition. **b** Levels of total proteins and electrophoretic bands obtained by densitometry of the dried strips. The results are expressed as mg protein (ml serum)^−1^ and the values are the mean ± SE of the mean of four animals for each experimental condition. *p < 0.05 indicates statistical difference from the controls and ^#^p < 0.05 from the monoarthritic condition

The serum protein profile was determined in the rats by means of cellulose acetate electrophoresis as well as the levels of plasma fibrinogen, a positive acute-phase protein, and serum transferrin, a negative acute-phase protein. Normally, serum cellulose acetate electrophoresis shows five protein bands, namely albumin, alpha-1, alpha-2, beta and gamma, as shown in Fig. 3. The total protein content was not different in controls, mono- and poly-arthritic rats. Furthermore, controls and monoarthritic rats presented the same protein bands. However, four protein bands were strongly modified by polyarthritis when compared to the control condition: albumin was 34 % lower and alpha-1, alpha-2 and gamma were 500, 380 and 260 % higher, respectively. Plasma fibrinogen levels were not different in control and monoarthritic rats, but they were 250 % higher in polyarthritic rats (Table 1). Transferrin levels were only slightly higher in polyarthritic rats (+15 %; Table 1).

**Table 1:** Levels of serum transferrin and plasma fibrinogen in control, mono- and poly-arthritic rats

Parameters	Groups		
	Control	Monoarthritis	Polyarthritis
Transferrin (mg dl^−1^)	91.00 ± 1.22	103.8 ± 3.32	114.8 ± 4.9*
Fibrinogen (mg dl^−1^)	1.88 ± 0.14	1.81 ± 0.09	6.56 ± 1.23^#,^*

Blood was collected from the inferior cava vein and processed as described in the “Methods” section. Values are mean ± SE of the mean of four animals for each experimental condition

* p < 0.05 indicates statistical difference from the controls and ^#^p < 0.05 from the monoarthritic condition

### Serum oxidative status

Serum oxidative status was evaluated by means of its total antioxidant capacity (TAC), protein thiols groups and protein carbonyl groups. The results are shown in Table 2. The TAC is expressed only in concentration units (μM), but the thiol and carbonyl groups are expressed as μM and, alternatively, as nmol mg^−1^, since they are protein associated parameters. The serum TAC was not different in control and monoarthritic rats, but it was 35 % lower in polyarthritic rats. The serum thiol and carbonyl groups were also not different in control and monoarthritic rats. However, the serum thiol groups were 51 % lower in the polyarthritic rats and the carbonyl groups were 40 % higher in the polyarthritic rats when compared with the controls.

**Table 2:** Total antioxidant capacity, levels of protein thiols and carbonyl groups in the serum of control, mono- and poly-arthritic rats

Parameter	Units	Groups		
		Control	Monoarthritis	Polyarthritis
Total antioxidant capacity	μM (eq. trolox)	708.1 ± 22.9	712.2 ± 40.4	471.3 ± 19.3^#,^*
Thiols groups	μM	230.9 ± 5.8	232.5 ± 17.2	118.8 ± 10.8^#,^*
	nmol mg^−1^ protein	4.41 ± 0.18	4.10 ± 0.30	2.33 ± 0.21^#,^*
Carbonyl groups	µM	302.6 ± 8.22	327.8 ± 14.2	426.0 ± 13.9^#,^*
	nmol mg^−1^ protein	5.60 ± 0.36	5.91 ± 0.30	7.89 ± 0.14^#,^*

The results of the total antioxidant capacity are expressed as µmol L^−1^ (μM) of trolox equivalents and the results of thiol and carbonyl groups are expressed as both μM and, alternatively, as nmol (mg protein)^−1^. Values are the mean ± SE of the mean of four animals for each experimental condition

* p < 0.05 indicates statistical difference from the controls and ^#^p < 0.05 from the monoarthritic condition

### Serum antioxidant components

The albumin, uric acid and ascorbate together represent more than 90 % of the total antioxidant activity of the serum (Erel 2004). Its levels are shown in Table 3. There was no difference in the levels of serum albumin between controls and monoarthritic rats, but polyarthritic rats presented lower serum albumin levels (−34 %). Serum uric acid was also not different between controls and arthritic animals, but it was 41 % higher in the polyarthritic condition. The levels of serum ascorbic acid were 23 and 35 % lower in mono- and poly-arthritic rats, respectively, when compared to the controls.

**Table 3:** The levels of serum antioxidant compounds in control, mono- and poly-arthritic rats

Parameters	Groups		
	Control	Monoarthritis	Polyarthritis
Albumin (μM)	328.8 ± 10.6	355.0 ± 6.2	218.2 ± 8.9*^,#^
Uric acid (μM)	78.5 ± 2.8	72.4 ± 6.3	110.6 ± 6.1*^,#^
Ascorbic acid (μM)	126.5 ± 8.5	98.2 ± 4.5*	82.9 ± 8.4*^,#^

Values are the mean ± SE of the mean of four animals for each experimental condition

* p < 0.05 indicates statistical difference from the controls and ^#^p < 0.05 from the monoarthritics

### Oxidative modifications in serum albumin

The contribution of albumin molecule to the serum oxidative status was evaluated by means of carbonyl groups, thiol groups and antioxidant capacity. The results are shown in Table 4. The results are also shown as percentage of the albumin-associated parameters in relation to the total serum parameters. This allows inferring the albumin contribution for each parameter. The contribution of albumin to the TAC was similar for all experimental groups (between 75 and 80 %). In absolute terms, the albumin antioxidant capacities of control and monoarthritic rats were not different, but that of polyarthritic animals was 34 % lower. This corresponds to the exact percentage of the serum albumin decrease caused by polyarthritis. The albumin thiols contents of control and monoarthritic rats were not different and they correspond to approximately 80 % of the serum total thiols for both conditions. In polyarthritic rats, however, the albumin thiols were approximately 71 % of the total thiols, and their absolute levels were 55 % lower than those found in the controls.

**Table 4:** Antioxidant capacity, thiol and carbonyl groups in the albumin molecule of control, mono- and poly-arthritic rats

Parameters	Units	Groups		
		Control	Monoarthritis	Polyarthritis
Albumin antioxidant capacity	μM (eq. trolox)	563.3 ± 52.0	531.2 ± 17.8	377.0 ± 7.0^#,^*
	% of TAC	80	75	80
Albumin thiols groups	μM	188.7 ± 9.2	181.6 ± 6.2	85.1 ± 6.2^#,^*
	% total thiol	82	78	71
	nmol/mg albumin	3.88 ± 0.27	3.33 ± 0.22	2.82 ± 0.18*
Albumin carbonyl groups	µM	92.3 ± 6.4	151.7 ± 3.2*	186.1 ± 7.6*^,#^
	% total carbonyls	30	36	36
	nmol/mg albumin	2.20 ± 0.04	2.78 ± 0.13*	5.93 ± 0.60^#,^*

The serum albumin separation was performed using affinity chromatography with Cibacron Blue 3G^®^ as described in the “Methods” section. The results are expressed in three ways: as μM, as nmol (mg albumin)^−1^ and as the percentage of albumin parameters in relation to the total parameters before chromatography. Values are the mean ± SE of the mean of 3–4 samples for each experimental condition

* p < 0.05 indicates statistical difference from the controls and ^#^p < 0.05 from the monoarthritic condition

**Fig. 4:**
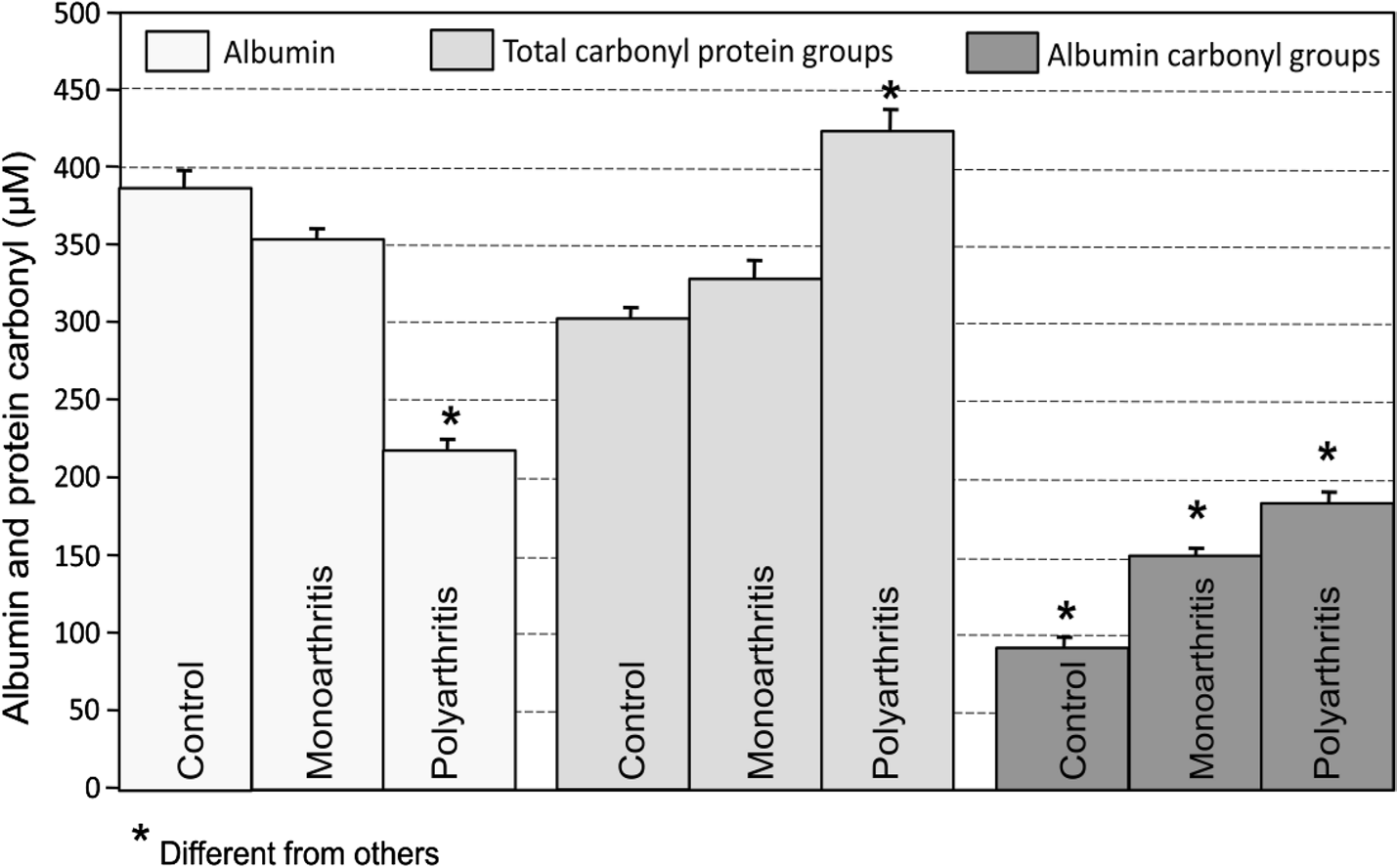
The contribution of the albumin carbonyl groups for the total carbonyl groups in the serum of rats with mono- and poly-arthritis. Values are expressed as µM and represent those shown in Tables 2, 3 and 4, respectively, for total serum carbonyl groups, serum albumin levels and albumin carbonyl groups. *p < 0.05 when a given value differs significantly from the others

The contribution of the albumin carbonyl groups to the total protein carbonyl groups can be best appreciated by examining Fig. 4 in conjunction with Table 4. In spite of the fact that the albumin concentration was decreased only in polyarthritis, its relative contribution to the total carbonyl groups was somewhat increased by both mono- and poly-arthritis (from 30 to 36 %; Table 4). This reflects the much more pronounced increase in the absolute contribution of albumin, whose carbonyl groups were 64 and 102 % higher in mono- and poly-arthritic animals, respectively.

## Discussion

Monoarthritis is considered a mild arthritis model in rats, which shows an inflammatory response in only one of the paws. Systemic effects are also present but normally mild, mainly changes in the liver metabolism (Bracht et al. 2012; Donaldson et al. 1993). The results of the present work showed that monoarthritic animals presented an inflammatory response that evolved only in the injected paw, but without weight loss and no changes of serum protein profile when compared to the controls. Thus, these parameters should be unsuitable for evaluating the systemic involvement of arthritis. On the other hand, polyarthritis is considered a severe arthritis model in rats, which shows a widespread inflammatory response, cachexia and systemic involvement (Comar et al. 2013; Donaldson et al. 1993). Our results showed that polyarthritic rats developed an intense inflammatory response also in the contralateral paw and furthermore strong changes in the serum protein profile: decreased albumin, increased globulins (alpha-1, alpha-2 and gamma bands) and increased plasma fibrinogen. The changes in the serum protein profile are mainly related with the induction of acute-phase proteins in the liver during systemic inflammation, which result in the increase in the levels of positive acute-phase proteins, such as fibrinogen, alpha-2 macroglobulin, C-reactive proteins, complement factors and others. Concomitantly, a decrease in the levels of the negative acute-phase proteins occurs, especially albumin. Similar results regarding the serum protein pattern were already reported by previous work with both adjuvant-induced rats and exacerbated rheumatoid arthritis in humans (Rothkopf-Ischebeck 1980; Sharp et al. 1982). Thus, the serum protein profile that was characterized here, strongly correlates with severe arthritis, but it was not sensible enough to differentiate mild arthritis from the healthy state.

Concerning the oxidative state of the serum, protein carbonyl groups were assessed as pro-oxidant status markers and the TAC, thiol groups, albumin, uric acid and ascorbic acid as antioxidant status markers. Before discussing the observations about the oxidative status of the various experimental groups, two technical aspects deserve preliminary comments: (a) the difference between thiol groups and albumin levels as serum antioxidant parameters and (b) the components of the serum TAC. Concerning (a), it should be remarked that the levels of free cysteine and GSH are minimal in the serum and, consequently, the thiol groups present in significant amounts are practically only those associated to proteins (Moriarty-Craige and Jones 2004; Oettl and Stauber 2007). As previously reported, the cysteine-34 of the albumin is the main source of thiol groups in the serum (70–80 %) and, thus, the antioxidant activity due to the serum thiol groups may be attributed mainly to albumin (Taverna et al. 2013). On the other hand, the serum thiol groups cannot be taken in place of albumin as an antioxidant parameter because other sites in this protein also react and sequestrate reactive species, mainly the six residues of methionine (Met-23, -87, -329, -298, -446 and -548) (Fanali et al. 2012). Thus, albumin is considered the main antioxidant in the serum but the thiol groups are only a portion of this larger antioxidant component. With reference to the TAC [point (b) above] it is usually expressed as μM, similarly to the serum antioxidant constituents. However, it is important to note that this may not represent the sum of the individual antioxidant constituents because their reactions display different stoichiometries. The method used for the determination of the TAC is based on the capacity of the serum to neutralize the ABTS·^+^ radicals and the results are expressed as trolox equivalents (Erel 2004). One trolox molecule receives one pair of electrons, becoming able to neutralize two ABTS·^+^ radicals. Thus, one equivalent of trolox represents one pair of electrons, but one equivalent of albumin may represent several electrons from many ABTS·^+^ because it bears many antioxidant sites in the same molecule.

Returning to the effects of adjuvant arthritis on the serum oxidative status (Tables 2, 3), it is possible to note that, ascorbic acid was the only parameter whose concentration was found to be modified by monoarthritis. The ascorbic acid concentration, in principle at least, can be regarded as an indicator for monoarthritis in as much as polyarthritis caused a further drop in the ascorbic acid concentration, combined with substantial modifications in other parameters. It is important to mention, however, that the ascorbic acid levels may also fluctuate according to the nutritional status. This fact weakens substantially the role of the ascorbic acid concentration as an indicator of mild arthritis unless it is combined with another indicator as, for example, the albumin carbonyl groups (see next paragraph).

The oxidative status of the serum albumin was analyzed on terms of three parameters: its antioxidant capacity, the presence of thiol groups and the presence of carbonyl groups. These measurements allowed to evaluate the contribution of this protein to the oxidative state of the serum. Albumin was responsible for approximately 75–80 % of the TAC and 80 % of the total thiol groups in control and monoarthritic rats. Similar values were also reported for serum albumin in healthy humans (Taverna et al. 2013; Oettl and Stauber 2007). On the other hand, the albumin antioxidant capacity and the albumin thiol groups were lower in polyarthritic rats. The decreased albumin antioxidant capacity is more likely the consequence solely of the decrease in the albumin concentration because both diminished equally (−34 %). However, the albumin thiol groups in polyarthritis were approximately half that of the controls and, thus, are more likely the consequence of both the diminished serum albumin levels combined with more oxidized levels of the sulfhydryl groups. Although these two parameters strongly correlate with severe arthritis, they are obviously no indicators for monoarthritis because no significant differences were found between healthy and monoarthritic rats.

The albumin carbonyl groups were only 30–36 % of the serum total carbonyl groups under all conditions, suggesting that the other serum proteins are equally or even more carbonylated than albumin. However, in absolute terms, the albumin carbonyl groups were significantly different in control, mono- and poly-arthritic rats. Thus, as ascorbic acid the serum albumin carbonyl groups may also be regarded as possible indicators of the severity of arthritis. Moreover, the changes in the serum levels of ascorbic acid and albumin carbonyl groups appeared in monoarthritis before the other systemic modifications that were evaluated here, such as the inflammatory response in the contralateral paw and the serum protein profile.

The results of the present work are in part innovative since they show that the levels of serum ascorbic acid and albumin carbonyl groups are differently changed in mono- and poly-arthritic rats. Thus, these data may be added to others that were previously characterized for evaluating the activity of rheumatoid arthritis, such as the serum protein profile and the C-reactive protein (Rothkopf-Ischebeck 1980; Sharp et al. 1982) and should also allow extrapolations for the serum of patients with greater or lesser severity of rheumatoid arthritis. However, it is important to highlight some limitations of this work, which used only male rats. As rheumatoid arthritis is more frequent in females, new approaches using simultaneously male and female rats should be encouraged. This would allow to evaluate the effect of the gender on these oxidative parameters.

## Conclusion

In summary, it can be said that the results of the present study revealed that (1) the serum oxidative state is strongly changed in the rats with adjuvant-induced polyarthritis; (2) both serum albumin levels and its oxidative state are also strongly changed by polyarthritis; (3) the levels of ascorbic acid and albumin carbonyl groups may be an useful indicator of the severity of rheumatoid arthritis in humans since they were different in control, mono- and poly-arthritic rats; and (4), the serum ascorbic acid concentration and the levels albumin carbonyl groups are quite sensitive indicators of mild arthritis since they appeared before other symptoms, such as systemic inflammation and modifications in the serum protein profile. If these findings are applicable or not to patients is a matter that depends on specific experiments at the clinical level that must demonstrate that the herein described oxidative changes also occur in rheumatoid arthritis.

## Methods

### Chemicals

Cibacron Blue 3G was purchased from GE Healthcare Life Sciences (São Paulo, SP, Brazil). Cellulose acetate strips for electrophoresis and cellulose membrane for dialysis were purchased from Inlab (Alamar Tecno-Científica Ltda, Diadema, Brazil). 6-Hydroxy-2,5,7,8-tetramethylchroman-2-carboxylic acid (Trolox)and the complete Freund’s adjuvant were purchased from Sigma Chemical Co (St Louis, MO, USA). Transferrin FS was purchased from DiaSys Diagnostic Systems GmbH (Holzheim, Germany). Commercial kits for the assay of albumin, uric acid and total proteins were purchased from Gold Analisa Diagnóstica Ltda (Belo Horizonte, MG, Brazil). All other chemicals were of analytical grade.

### Animals and treatments

Male *Holtzman* rats weighing 180–210 g (about 50 days old) were used for induction of adjuvant arthritis. Polyarthritis was induced by injection in the left hind paw of 0.1 ml of Freund’s adjuvant containing 5.0 mg ml^−1^ of heat inactivated *Mycobacterium tuberculosis*, derived from the human strain H37Rv, suspended in mineral oil (Pearson and Wood 1963). Monoarthritis was induced in the same manner but with 0.1 ml of Freund’s adjuvant containing only 1.0 mg ml^−1^ of *M. tuberculosis* (Sigma-Aldrich, St Louis, MO, USA) (Bracht et al. 2012). Rats of similar ages served as controls. After 18 days of the adjuvant injection, rats were selected for the experiments. All procedures were done in accordance with the world-wide accepted ethical guidelines for animal experimentation and previously approved by the Ethics Committee for Animal Experimentation of the University of Maringá (Protocol 094/2013-CEEA).

### Blood collection and preparation

Rats fasted for 12 h were anesthetized with intraperitoneal injection of sodium pentobarbital (50 mg kg^−1^) and the peritoneal cavity was surgically exposed. Blood was then collected from the cava vein and poured in tubes either with 110 mM sodium citrate (BD vacutainer tube^®^) for plasma or without anticoagulant for serum. After centrifugation at 3000*g* for 10 min, the supernatant was separated as serum or plasma fraction.

### Profile of serum proteins

Protein electrophoresis was performed with cellulose acetate strips (2.5 × 14 cm; Cellogel Electrophoresis Co.) and protein fractions were quantified by densitometry. Briefly, the cellulose acetate strips were previously soaked for 10 min into 60 mM sodium barbital buffer (pH 8.6) and thereafter placed on the bridge of a horizontal electrophoresis tank. Then, the serum was applied on each strip at the cathodal end, a 3.0 mA current was applied at 250 V and electrophoresis was run for 30 min. After electrophoresis the strips were soaked into Ponceau S red dye for 10 min and destained with glacial acetic acid. The strips were dried on a kiln at 70 °C and the quantification of the electrophoretic bands was made with a densitometer (CELM DS-35^®^) on blue filter for Ponceau S.

The serum transferrin was measured by immunoturbimetry and the values expressed as mg dl^−1^. The fibrinogen was measured in the plasma by coagulometry using a commercial kit (TriniClot™ Fibrinogen Kit) and the results expressed as mg dl^−1^. Total proteins and albumin levels were measured spectrophotometrically using commercial kits.

### Purification of serum albumin

Each sample consisted of a serum *pool* that was obtained from three animals and the albumin separation was performed by affinity chromatography as described previously (Guerin-Dubourg et al. 2012). Briefly, the affinity column of Cibacron Blue 3G^®^ was built on a plastic syringe (1.3 cm of diameter and 8.2 cm of height), where the gel was packed to a final volume of 10 ml. The column was then equilibrated with 50 mM Tris–HCl buffer (pH 7.4) and 4.0 ml of serum sample was applied. After, the column was washed with TRIS–HCl buffer. In this process, albumin is retained and the other proteins are eluted. The albumin was then eluted with 1.5 M NaCl, which was dialyzed with cellulose membrane (12–16 kDa of cut-off and 25 Å of porosity) against 0.1 M PBS buffer (pH 7.4) at 4 °C for 24 h under gentle agitation. The dialysate was adjusted to a final volume of 5.0 ml with PBS buffer for measuring thiol groups, carbonylated proteins and albumin.

### Total antioxidant capacity (TAC)

The TAC of the serum was measured by spectrophotometry using 2,2′-azino-bis(3-etylbenzthiazoline-6-sulphonic acid) or ABTS (Erel 2004). The antioxidant activity was calculated from the standard curve prepared with trolox (6-hydroxy-2,5,7,8-tetrametylchloraman-2-carboxylic acid), a water-soluble analog of vitamin E, and the results were expressed as nmol (ml serum)^−1^.

### Total and albumin carbonyl and sulfhidryl groups (thiols)

The thiol content was measured by spectrophotometry (412 nm) using DTNB (5,5′-dithiobis 2-nitrobenzoic acid) and the contents were calculated using the molar extinction coefficient (ε) of 1.36 × 10^4^ M^−1^ cm^−1^ (Faure and Lafond 1995). The protein carbonyl groups contents were measured by spectrophotometry (370 nm) using 2,4-dinitrophenylhydrazine (DNPH) and the contents were calculated using the molar extinction coefficient (ε) of 2.2 × 10^4^ M^−1^ cm^−1^ (Levine et al. 1990). The values of both carbonyl and thiol groups were expressed as nmol of carbonyl/thiol groups (ml serum)^−1^ or, alternatively, nmol (mg protein)^−1^.

### Ascorbic acid and uric acid

Uric acid was measured by an enzymatic method using commercial kits (Gold Analisa^®^) and the results were expressed as mg (ml serum)^−1^. Ascorbic acid was measured by spectrophotometry (520 nm) with the 2,4-DNPH-thiourea-CuSO_4_ (DTC) reagent (Omaye et al. 1979). The contents were calculated using a standard curve prepared with ascorbic acid and the expressed as mg (ml serum)^−1^.

### Statistical analysis

The error parameters presented in graphs and tables are standard errors of the means. Statistical analysis was done by means of the GraphPad Prism Software (version 5.0). The statistical significance was analyzed by means of one-way ANOVA and the Newman–Keuls multiple comparisons test was applied with the 5 % level (p < 0.05).
